# Unintended effects of a targeted maternal and child nutrition intervention on household expenditures, labor income, and the nutritional status of non-targeted siblings in Ghana

**DOI:** 10.1016/j.worlddev.2018.02.025

**Published:** 2018-07

**Authors:** Katherine P. Adams, Travis J. Lybbert, Stephen A. Vosti, Emmanuel Ayifah, Mary Arimond, Seth Adu-Afarwuah, Kathryn G. Dewey

**Affiliations:** a University of California, Davis, Program in International and Community Nutrition, Department of Nutrition, One Shields Avenue, Davis, CA 95616, United States; b University of California, Davis, Department of Agricultural and Resource Economics, One Shields Avenue, Davis, CA 95616, United States; c University of Mannheim, Chair of Econometrics/Center for Evaluation and Development, Department of Economics, L 7, 3-5, Room 131, D-68131 Mannheim, Germany; d University of Ghana, Department of Nutrition and Food Science, P.O. Box LG 134, Legon, Accra, Ghana

**Keywords:** Intrahousehold spillovers, Randomized trial, Expenditures, Income, Child nutrition

## Abstract

It is common for health and nutrition interventions to target specific household members and for evaluations of their effects to focus exclusively on those members. However, if a targeted intervention changes a household’s utility maximization problem or influences decision-making, households might respond to the intervention in unintended ways with the potential to affect the wellbeing of non-targeted members. Using panel data from a randomized controlled nutrition trial in Ghana, we evaluate household behavioral responses to the provision of small-quantity lipid-based nutrient supplements (SQ-LNS) to mothers and their infants to prevent undernutrition. We find that targeted supplementation with SQ-LNS had a positive effect on household expenditures on food, including some nutrient-rich food groups, as well as on non-food goods and services. We also find a positive impact on labor income, particularly among fathers. We then explore intrahousehold spillover effects on the nutritional status of non-targeted young children in the household. We find evidence that the targeted provision of SQ-LNS led to higher height-for-age z-scores among non-targeted children in the LNS group compared to the non-LNS group, though only among those with relatively taller mothers, which is an indicator of a child’s growth potential. These findings support existing evidence and suggest that unintended behavioral responses and spillover are a real possibility in the context of nutrition interventions targeting nutritionally-vulnerable household members. Thoughtfully considering this possibility in the design, analyses, and evaluation of targeted nutrition interventions may provide a more complete picture of overall effects.

## Highlights

• The targeted provision of maternal and infant SQ-LNS affected household behavior.
• Targeted SQ-LNS had a positive effect on household food and non-food expenditures.
• Higher labor income may have permitted higher expenditures.
• We find evidence of positive nutritional spillovers onto some non-target children.
• Targeted interventions may affect the wellbeing of non-target household members.

## 1 Introduction

Nutrition in the earliest stages in the life-cycle – from conception through a child’s second birthday – shapes a child’s growth trajectory and developmental potential and, as such, has long-term consequences for human capital acquisition and economic productivity in adulthood (Black et al., 2013, Grantham-McGregor et al., 2007, Hoddinott et al., 2013, Victora et al., 2010, World Bank, 2006). This early, pivotal period in the life-cycle has therefore become the focus of many maternal and child nutrition interventions providing, e.g., conditional cash, health and nutrition information, or supplementation to mothers and/or young children (Ainsworth and Ambel, 2010, Bhutta et al., 2013). Evaluations of the efficacy or effectiveness of these interventions logically center around estimates of their effects on the nutrition, health, and development of the targeted household member(s). But household behavior is not static, and if a targeted intervention introduces changes to a household’s utility maximization problem in the form of new information or changes in constraints or relative prices, or if it influences the household decision-making process, the intervention may induce behavioral responses outside the scope of those intended. The potential implications of such behavioral responses include intrahousehold spillovers that may affect the wellbeing – either positively or negatively – of *non*-targeted household members.

This study explores household behavioral responses to and intrahousehold spillover effects associated with the targeted provision of small-quantity lipid-based-nutrient-supplements (SQ-LNS), which are food-based home fortificants designed to enhance the diets of women and young children by providing a wide range of micronutrients along with some key macronutrients (Arimond et al., 2015). SQ-LNS were provided to mothers during pregnancy and the first six months postpartum and to their infants from 6 to 18 months of age as part of a randomized controlled trial in Ghana designed to test their efficacy vis-à-vis maternal multiple micronutrient capsules and iron-folic acid capsules. Using socioeconomic data collected during the randomized trial, we find a positive effect of targeted supplementation with SQ-LNS on *per capita* household expenditures on food and non-food goods and services. We then consider whether the intervention had an effect on the labor income of SQ-LNS households, which could have permitted higher expenditures. Although we find no difference in the labor income of the target mothers who were participating in the trial, we find evidence suggesting a positive impact on total household labor income *per capita* as well as on the labor income of the husbands/partners of target mothers.

Depending on intrahousehold resource allocation, higher household expenditures on food induced by the targeted intervention had the potential to influence the nutritional status of non-targeted household members. We use anthropometric data, which were collected at several time-points during the trial, on the youngest sibling^1^ under age five to explore this potential spillover effect. While we find no overall effect of the targeted provision of SQ-LNS on the siblings’ z-scores of height-for-age, weight-for-age, or BMI-for-age, we do find evidence of a positive effect on height-for-age z-scores among siblings with relatively tall mothers when the mother-infant pair received SQ-LNS.

Together, these findings contribute to a small but growing body of literature evaluating spillover effects of targeted maternal and child health and nutrition interventions in developing countries (Adhvaryu and Nyshadham, 2014, Fitzsimons et al., 2016, Kazianga et al., 2014). The results presented in this study, together with the previous findings in the literature, underscore the value of assessing not only the effects of an intervention on targeted household members but also in collecting data to facilitate an assessment of how households respond to such interventions and whether those responses generate intrahousehold spillovers.

The remainder of the paper is organized as follows: we begin with background information on SQ-LNS and the randomized controlled trial. We also present a brief review of relevant literature to set our study within the context of previous work on intrahousehold spillovers. This is followed by a description of the data used in the analyses, our empirical strategy, and the results. Finally, we posit several mechanisms through which the behavioral responses may have been generated, present limitations of our findings, and make concluding remarks.

## 2 Background

Ready-to-use therapeutic foods (RUTF) are fortified, lipid-based food products that are currently widely used in the treatment of children with severe acute malnutrition (World Health Organization, World Food Programme, United Nations System Standing Committee on Nutrition, & United Nations Children's Fund, 2007). The success of these therapeutic products, which are energy-dense and consumed in large quantities over a relatively short period of time for rehabilitative purposes, has spurred the development of similar products, collectively called small-quantity lipid-based nutrient supplements (SQ-LNS), to *prevent* undernutrition. Compared to the therapeutic products, SQ-LNS are administered at a much lower daily ration (typically 20 g/day, ∼118 kcal/day) but with a higher concentration of micronutrients (Arimond et al., 2015, Dewey and Arimond, 2012). SQ-LNS typically contain vegetable oil, dried skimmed milk, peanut paste, sugar, and a vitamin-mineral mix, and because the micronutrients in SQ-LNS are embedded in a food base, the supplements also provide some macronutrients (fats, protein, and carbohydrates). As described next, the efficacy of SQ-LNS was recently evaluated in a randomized controlled trial in Ghana.

### 2.1 Description of the randomized trial

From December 2009 through March 2014, the International Lipid-Based Nutrient Supplement (iLiNS) Project^2^ administered a targeted randomized controlled trial in Ghana to evaluate the efficacy of a duo of SQ-LNS products designed for maternal consumption during pregnancy and the first six months postpartum and for consumption in early childhood to prevent undernutrition. The trial was approved by the ethics committees of the University of California, Davis, the Ghana Health Service, and the University of Ghana Noguchi Memorial Institute for Medical Research and was registered at clinicaltrials.gov as NCT00970866. All study participants provided informed consent.

The catchment area for recruitment of pregnant women into the trial was situated along a busy commercial corridor in the Lower Manya Krobo and Yilo Krobo districts in the Eastern Region of Ghana. Because of its proximity to the Volta River and Lake Volta, fish is a component of the staple diet in the area, along with maize, cassava, and, to a lesser extent, leafy vegetables (Adu-Afarwuah et al., 2015). The communities along the corridor are linked by a robust public transportation system, so households have reliable access to the large, twice-weekly market and other smaller markets in the area. Rates of maternal and early childhood undernutrition in this region of Ghana are, in general, comparable to national rates. In 2011 among all children under age five in the Eastern Region, the average height-for-age z-score (HAZ) was -0.9 and 21.3% were stunted (HAZ <−2 SD) (Ghana Statistical Service, 2011a). The average weight-for-age z-score (WAZ) among this population was −0.7 in 2011, with 10% classified as underweight (WAZ <−2 SD). Approximately 46.2% of children 6–59 months old in the Eastern Region were anemic in 2011 (Ghana Statistical Service, 2011b), and the rate of anemia in women of childbearing age was 58.3% in 2008 (Ghana Statistical Service, 2009).

Recruitment and enrollment of pregnant women into the trial was done on a rolling basis from December 2009 to December 2011. Women attending antenatal clinics at one of the four main health facilities in the area were approached for potential participation in the trial,^3^ and interested women were then screened to determine eligibility.^4^ Eligible and willing women (*n* = 1320) were formally recruited into the study and randomized at the individual level into one of the trial’s three equally-sized arms in which women received either (1) daily iron-folic acid capsules throughout pregnancy, a component of the current standard of antenatal care in Ghana, and a placebo (low-dose calcium capsule) during the first six months postpartum (IFA group), (2) daily multiple micronutrient capsules during pregnancy and the first six months postpartum (MMN group), or (3) SQ-LNS during pregnancy and the first six months postpartum (LNS group); from 6 to 18 months of age, the infants of women randomized into the LNS group also received an SQ-LNS-product designed for children. The infants of women randomized into the IFA or MMN groups did not receive any supplementation. The nutrient compositions of the SQ-LNS product for women, the multiple micronutrient capsules, and the iron-folic acid capsules are reported in Adu-Afarwuah et al. (2015), and the nutrient composition of the SQ-LNS product for child consumption is reported in Adu-Afarwuah et al. (2016).

At enrollment, each woman received instructions on how to take her assigned supplement and was given the following nutrition message (the same message was provided to all women in the trial regardless of treatment group): “Do not forget to eat meat, fish, eggs, fruits and vegetables whenever you can. You still need these foods even if you take the supplement we have given you.” During pregnancy and the first six months postpartum, all women in the trial, regardless of treatment group, were visited by project staff every two weeks to deliver supplements and collect data on morbidity and adherence to the study protocol. The nutrition message was repeated to all women at a laboratory visit at 36 weeks of gestation. After the infants were born, staff made weekly home visits to deliver supplements (if applicable) and collect morbidity and adherence data on all infants. A message about the importance of feeding the infant diverse, nutritious foods as well as continuing to breastfeed was also communicated to each woman when her infant was six months old. Beyond delivery of different supplements, the frequency of contact with the households by iLiNS staff and the content of those visits were, by design, uniform across the treatment groups. And while the individual-level randomization meant households in the group that received SQ-LNS could have been living very near households in the group that did not receive SQ-LNS, the likelihood (and self-reported frequency^5^) of sharing of supplements at any meaningful level between groups was low given the small daily dosage and the frequency of contact with study staff who were reiterating messages discouraging sharing.

In evaluating the efficacy of SQ-LNS, which was the primary objective of the randomized trial but is not the subject of the present analysis, the main outcomes of interest were birth size and attained growth of the target child by 18 months. Adu-Afarwuah et al. (2015) showed that among this sample in Ghana, providing women with SQ-LNS during pregnancy increased average birth weight relative to women who received iron-folic acid capsules, though there was no difference in birth weight relative to the women who received multiple micronutrient capsules. The analyses in Adu-Afarwuah et al. (2015) also demonstrated statistically significant heterogeneity in the effect of SQ-LNS on birth outcomes by parity. Among first-time mothers, the provision of SQ-LNS compared to iron-folic acid capsules and compared to multiple micronutrient capsules had a large impact on birth weight, length and head circumference and decreased the incidence of low birth weight (birth weight <2500 grams), whereas there was no effect in multiparous mothers. By 18 months of age, the attained length and weight of children in the LNS group were greater compared with those of children in the IFA or MMN group, which reflected the observed differences at birth (Adu-Afarwuah et al., 2016). While this improvement in attained size was evident in the whole sample of children, it was more pronounced in some subgroups, including children with taller mothers (in particular, consumption of SQ-LNS decreased the probability of stunting among children with relatively tall mothers but not among those with shorter mothers). The analyses presented in this paper broaden the scope of potential effects of the intervention by exploring household behavioral responses to it and the nutritional status of non-targeted siblings.

### 2.2 Behavioral responses and spillover effects

Our study fits into a nascent body of literature that has provided evidence of behavioral responses to maternal and child health and nutrition interventions in African contexts along margins beyond those directly targeted by the intervention, sometimes generating measurable intrahousehold spillovers. One such study is Adhvaryu and Nyshadham (2014) who found that in Tanzania, exposure to a prenatal iodine supplementation program changed parental investment behavior. In particular, children who were exposed to the iodine supplementation program *in utero* had a higher probability of later being vaccinated against polio, diphtheria, and measles. The study also found evidence of an intrahousehold spillover effect; older siblings of the infants exposed to iodine supplementation were also more likely to be vaccinated, which the authors attributed to a reallocation of resources among siblings stemming from a parental preference for equity among their children. We likewise find evidence suggesting the possibility that households may have changed their behavior in an effort to improve the nutrition of household members who were not directly benefitting from the intervention.

Two other studies are similar in spirit. First, Fitzsimons et al. (2016) evaluated household behavioral responses to a targeted cluster-randomized intervention in Malawi that provided information on infant feeding practices^6^ to mothers with a child under six months of age. The study found that in treated households, the diets of young children improved, as did overall household consumption of proteins, fruits, and vegetables. The study also found evidence that the provision of information about child nutrition may have induced fathers to increase their labor supply, presumably to fund higher food consumption. In another study, Kazianga et al. (2014) evaluated the effects of two school-feeding programs on the nutritional status of preschool-aged siblings in rural Burkina Faso. A year into a ‘take home rations’ school feeding program,^7^ the authors found a positive effect on the weight-for-age z-scores (WAZ) of preschool age siblings of children in treated schools, although height-for-age z-scores (HAZ) were unaffected. The other school feeding program, school meals, had no effect on the WAZ or HAZ of the younger siblings. The authors attributed the spillover effect of the take home rations program to intrahousehold food redistribution, which may have been more easily achieved under the take-home rations program relative to school meals.

Our study contributes new evidence to this emerging body of literature in the context of an intervention in Africa that, to our knowledge, has not previously been studied. Unlike the nutrition information intervention in Fitzsimons et al. (2016) in which the intended behavioral response was an improvement in dietary quality, and unlike the targeted intervention in Kazianga et al. (2014) in which the quantity of the food transfer was large enough that intrahousehold reallocation might lead to spillovers, we consider behavioral responses and spillovers induced by simply providing mothers and their infants with food-based supplements that they were instructed to mix with their everyday food. The daily rations of LNS were small in quantity (118 kcal/day), and the brief nutrition messages that were repeated on occasion throughout the intervention were uniform across groups. As such, it is unlikely that the transfer of SQ-LNS or the nutrition information alone would have created a condition in which intrahousehold spillovers were possible. Rather, unintended changes in household behavior – here changes in spending patterns and labor income – are signals of an interconnectedness among household members, and a willingness and ability to make marginal changes in behavior, that made spillovers a possibility. Combined with the evidence in the literature, our findings suggest that unintended behavioral responses and spillovers are a real possibility in the context of targeted nutrition interventions and that thoughtfully considering this possibility in the design, analyses, and evaluation of targeted nutrition interventions may provide a more complete picture of the effects of an intervention.

## 3 Data

### 3.1 Outcome variables

The household expenditure data used in this study were gleaned from an expenditures questionnaire used to ask the household member primarily responsible for food preparation and meals to recall food expenditures (based on a 1-week recall period), frequent non-food expenditures (1-month recall), and infrequent non-food expenditures (12-month recall). A randomly selected subsample of approximately 55% of participating households was selected to provide data on household expenditures. For this analysis, we further subdivide food expenditures into “nutrient-rich food groups” which are defined as those foods that are good sources of high-quality protein and/or micronutrients that may be low in diets dominated by starchy staple foods. These include animal-source foods, fruits (excluding plantains), vegetables (excluding starchy vegetables such as cassava and yam), pulses, and nuts.

Labor income data for each household member were scheduled to be collected from the full sample of households participating in the trial at three time points after maternal randomization. The questionnaire respondent, who was the target mother participating in the randomized trial, was asked to report the income each household member typically receives from his/her primary work. From the household roster of income information, we focus on the effects of the targeted intervention on the labor income of the target mother who directly participated in the trial, her husband,^8^^,^^9^ and total household labor income per capita. For reference, the most common occupation among the target women in the sample was petty trade (48%), while approximately 20% reported no labor income, and just 1% identified farming as their primary work. The husbands of target mothers were drivers/drivers’ assistants (20%), artisans (primarily self-employed carpenters and masons) (19%), shop owners (8%), mechanics (7%) and teachers (6%).

Finally, to assess intrahousehold spillovers, we use anthropometric data on the youngest sibling under age five in the household that were collected at two time points after maternal randomization. The criteria for inclusion in the sibling subsample were that the child shared the same biological mother as the target infant and that s/he was less than 60 months of age at maternal enrollment into the trial. In our analysis we use three measures of sibling nutritional status: height-for-age, weight-for-age, and BMI-for-age. We constructed z-scores of the anthropometric measures, which enables the comparison of an individual child’s anthropometric measurements (length/height and weight) to children in the reference population (O’Donnell, Doorslaer, Wagstaff, & Lindelow, 2007).^10^^,^^11^ Approximately 28% of the siblings were stunted at first measurement, higher than the rate of stunting in the Eastern Region of Ghana among children under five, which was 21.3% in 2011 (Ghana Statistical Service, 2011b).^12^ Rates of underweight at first measurement were similar to the 2011 regional rate: 11% in the sibling sample compared to 10.5% in the Eastern Region. Less than 2% of the siblings would be considered wasted or thin according to their BMIZ, while at the other end of the spectrum, less than 1% of the siblings would be classified as obese.

**Fig. 1:**
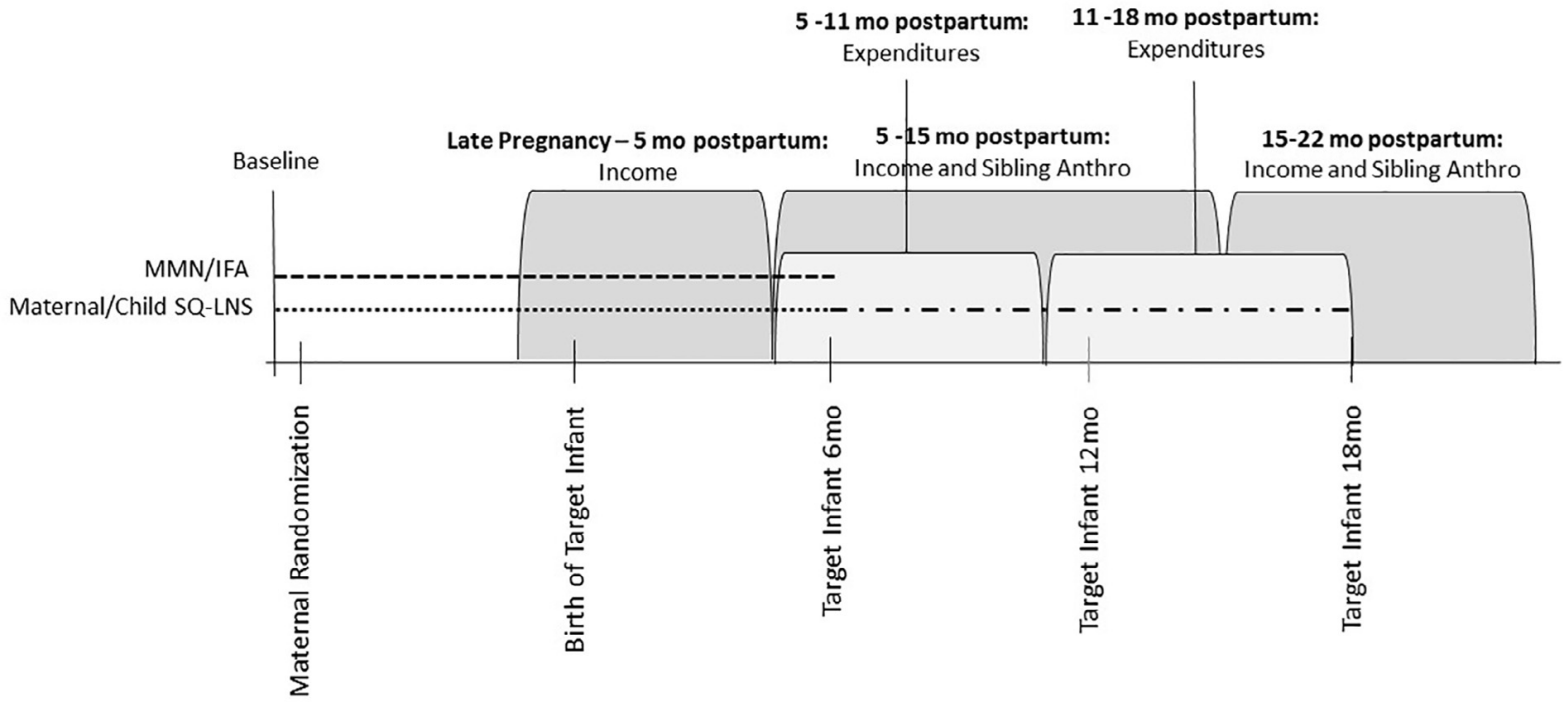
Timeline of data collection notes: maternal/child SQ-LNS refers to households in which the mother received SQ-LNS through pregnancy and the first six months postpartum (marked by the dotted line) and then her infant received SQ-LNS from 6 to 18 months postpartum (marked by the dash-dotted line). MMN/IFA refers to households in which the mother received multiple micronutrient capsules/iron-folic acid capsules through pregnancy and the first six months postpartum (and her infant received no supplementation), marked by the dashed line.

As detailed in the Online Appendix, although data collection was scheduled to occur at approximate dates relative to maternal enrollment into the trial and, after the birth of her infant, relative to the infant’s age, the actual timing of data collection used to construct our outcome variables varied substantially across households. As a result, we organize each round of data collection by intervals, where each interval is defined relative to the birth of the target infant.^13^^,^^14^ The timeline in Fig. 1 shows the intervals of data collection for each outcome variable. Households were visited once to collect income data during the interval from late pregnancy until five months postpartum. Households were again visited between 5 and 15 months postpartum to collect income data. Households with a child in the sibling subsample were also visited during this interval to collect anthropometric data. Final rounds of income and sibling anthropometric data collection occurred between 15 and 22 months postpartum. By design to reduce respondent fatigue, household expenditure data were collected at intervals slightly offset from income and anthropometric data collection. Household expenditure data were collected once during the interval from 5 to 11 months postpartum and once during the interval from 11 to 15 postpartum. In Online Appendix Tables A5–A8 we confirm that our main results are robust to controlling for delays in enumeration, which are potentially endogenous.

### 3.2 Balance, attrition, and selection

Table 1 summarizes the background characteristics and reports differences in these characteristics by treatment group.^15^ Mothers randomized into the LNS group had a slightly lower and statistically significant (*p* = .05) probability of reporting using vitamin and mineral supplements during their pregnancy prior to enrollment into the trial. The groups were otherwise balanced among these characteristics, and using chi-square tests of joint significance, we fail to reject the null of joint orthogonality of the background characteristics in the full sample, the expenditures subsample, and the sibling subsample.

**Table 1:** Maternal and sibling background characteristics for full analytic sample.

Variable	Definition	N	Mean	Std. Deviation	P-value^*^
Maternal Age	Maternal age in years	1235	26.7	5.5	0.61
Maternal Education	Maternal years of education	1235	7.4	3.7	0.56
Children	Number of target mother’s living biological children	1235	1.2	1.2	0.55
Maternal Height	Maternal height in centimeters	1216	158.9	5.7	0.55
Maternal Gestational Age	Maternal gestational age at enrollment in weeks	1235	16.2	3.2	0.91
Maternal Supplement Use	= 1 if mother reported using vitamin and mineral supplements since becoming pregnant	1235	0.9	0.3	0.05
Electricity	= 1 if electricity is main source of household lighting	1235	0.9	0.4	0.45
Sibling Age	Age in months at maternal enrollment into trial	370	35.5	12.0	0.91
Sibling Female	= 1 if sibling is female	370	0.5	0.5	0.72
Maternal Height (Sibling Sub-sample)	Maternal height in centimeters among mothers with a child under age five at enrollment	370	159.2	5.6	0.63

^*^P-value for two-sided t-test of difference in means between SQ-LNS group and non-SQ-LNS group.

*Notes:* ‘N’ indicates the number of mothers for whom post-randomization income data are available. The sample size for ‘sibling’ variables is limited to households with a sibling who was under age five at maternal enrollment into the trial and for whom post-randomization anthropometric data are available.

We aimed to collect post-randomization expenditures data from 736 households, income data from the full sample of 1320 households, and sibling anthropometric measures from 436 siblings. Achieved sample sizes at each interval of post-randomization data collection were well below the targets: for expenditures, n = 588 at the 5–11 months postpartum interval of data collection, and n = 545 at the 11–18 months postpartum interval; for income, n = 1179 at the late pregnancy – 5 months postpartum interval, n = 1058 at the 5–15 months postpartum interval, and n = 997 at the 15–22 months postpartum interval; for sibling anthropometry, n = 321 at the 5–15 months postpartum interval, and n = 306 at the 15–22 months postpartum interval. Attrition rates by intervention group at each interval of data collection are summarized in Table 2. Patterns of attrition include both intermittent missingness and permanent drop-out.^16^ With one exception,^17^ rates of attrition were not statistically different between groups. To further assess the comparability of the observed groups at each interval, we compared the set of background characteristics from Table 1 at each interval of data collection. The results in Online Appendix Table A3 show that the pre-enrollment difference in supplement use between the two groups remained evident at some intervals of data collection, but the two groups appear otherwise well balanced among all other background characteristics.

**Table 2:** Rates of attrition by time interval of post-randomization data collection and intervention group.

Survey	Intervention group	N	Time interval of post-randomization data collection		
			Late pregnancy – 5 mo postpartum	5–11 mo postpartum^a^|5–15 mo postpartum^b^	11–18 mo postpartum^a^|15–22 mo postpartum^b^
Household Expenditures	LNS	239		13.8%	22.6%
	Non-LNS	497		21.9%^***^	26.6%

Income	LNS	440	10.2%	18.4%	24.1%
	Non-LNS	880	10.5%	19.8%	23.1%

Sibling Anthropometry	LNS	144		25.0%	29.5%
	Non-LNS	292		26.0%	28.5%

Significance codes: ^***^(p < .01), ^**^(p < .05), ^*^(p < .1) indicate statistically significant difference in within-interval rate of attrition between groups.

*Notes:* Income data were collected from the full sample of households. Expenditure data were collected from a random subsample (approximately 55%) of households. The sample size for ‘sibling’ variables is limited to households with a sibling who was under age five at maternal enrollment into the trial.

a Relevant intervals for household expenditures data collection.

b Relevant intervals for income and sibling anthropometric data collection.

Finally, although all women who attended one of the antenatal clinics during the recruitment period were screened for eligibility and, if eligible, offered enrollment, some eligible women declined enrollment. Table A4 in the Online Appendix compares the characteristics of women who were enrolled to women who declined enrollment into in the trial.^18^ On average, women who enrolled were younger than those who declined, but the two groups were otherwise similar, on average, in terms of education, number of living children, cell phone ownership, and native language.

## 4 Empirical strategy

As previously described, mothers and infants were randomized into three groups, one in which the mother and her infant received SQ-LNS, one in which the mother received multiple micronutrient capsules, and one in which the mother received iron-folic acid capsules. The effect of the intervention on the birth outcomes of target infants, described in Adu-Afarwuah et al. (2015), and on attained size by 18 months of age, described in (Adu-Afarwuah et al., 2016), are based on comparisons between the three groups. However, SQ-LNS are different from either form of capsule in a number of ways. First, the characteristics of the physical product itself are different, as unlike capsules, SQ-LNS provide a small amount of calories (∼118) and macronutrients (protein, fat, carbohydrates). Further, SQ-LNS are consumed like a food (either combined with other foods or consumed alone) compared to swallowing a capsule. The design of the randomized trial also distinguished SQ-LNS from the capsules in several ways. In particular, iron-folic acid supplementation during pregnancy is a component of the standard of antenatal care in Ghana, and women randomized into either of the capsule groups were blind to which capsule they were receiving. As such, women in the SQ-LNS group were being asked to consume something completely new to them, while the mode of supplementation of women in either capsule group was not outside the norm. Moreover, SQ-LNS were provided to children from 6 to 18 months as part of the trial, while the children of women randomized into eitherof the capsule groups did not receive any supplementation. Given these differences, we combine those who received either form of capsules into one group and compare them to those who received SQ-LNS. As shown in Online Appendix Tables A9–A12, key results are qualitatively very similar when we compare across the three groups.^19^

Identification of the causal effects of targeted maternal and infant provision of SQ-LNS relies on random assignment to the intervention groups. For each outcome variable, we have a panel of several post-randomization observations (three observations for labor income and two observations for expenditures and sibling anthropometry) and no baseline. Therefore, unlike the common difference-in-difference specification with one baseline and one follow-up observation, we simply estimate the average impact over the course of the intervention by pooling all post-randomization observations. Particularly for dependent variables with relatively low autocorrelation like income and expenditures, using multiple post-randomization observations, even without a baseline, can improve power (McKenzie, 2012).

For j=1,…,H households and i=1,2,3 intervals of data collection, we estimate the average effect of the provision of SQ-LNS to a mother and her infant on each outcome of interest using the following random effects specification (Rabe-Hesketh & Skrondal, 2012)

(1) $$y_{\mathrm{ji}}={\mathrm{LNS}}_{j}+\theta I_{\mathrm{ji}}+\delta X_{j}+\gamma E_{\mathrm{ji}}+\alpha_{j}+\varepsilon_{\mathrm{ji}}\text{,}$$

where $y_{\mathrm{js}}$, is an outcome for household j at interval i. ${\mathrm{LNS}}_{j}$ is an indicator variable equal to one if the mother-infant pair was randomized to receive SQ-LNS and zero otherwise. The intervals of data collection are indicated in the vector $I_{\mathrm{ji}}$. To improve the precision of our estimates, we also include $X_{j}$, a vector of baseline controls. For the expenditures and income specifications, these controls are maternal years of education, an indicator variable for household electrification, and a set of indicators for year of maternal enrollment into the trial. The baseline controls for sibling anthropometric outcomes include those in the income and expenditure specifications in addition to sibling age at maternal enrollment into the trial, sibling gender, maternal gestational age at enrollment, and z-score of maternal height. We also control for enumerator/anthropometrist effects with a set of indicator variables in the vector $E_{\mathrm{ji}}$. The parameter $\alpha_{j}$ is a household-level random effect, and $\varepsilon_{\mathrm{ji}}$ is an idiosyncratic error. To account for correlation in the error over time for a given household, we cluster the standard errors at the household level.^20^

We assess heterogeneity in the estimated effects of randomization to the LNS group using interactions between ${\mathrm{LNS}}_{j}$ and the covariate predicted to modify the effect of LNS on the outcome of interest.

## 5 Results

### 5.1 Effect on household expenditures

We begin with the effect of the provision of SQ-LNS to the target mother and infant on household expenditures. We report the estimated effects of SQ-LNS on the inverse hyperbolic sine (IHS) of household expenditures in Table 3.^21^ For reference, over the course of the intervention households in the non-LNS group were spending an average of $7.01 (2011 USD) per capita per week on food (standard deviation of $3.54), $3.71 on nutrient-rich foods (standard deviation of $2.21), $4.20 on frequently purchased non-food goods and services (standard deviation of $5.94), and $4.36 on infrequently purchased non-food items (standard deviation of $5.77).

**Table 3:** Effect of the provision of SQ-LNS to mothers and their infants on per capita weekly household expenditures.

	(1) Food	(2) Nutrient-Rich Food	(3) Frequent Non-Food	(4) Infrequent Non-Food	(5) Food	(6) Nutrient-Rich Food	(7) Frequent Non-Food	(8) Infrequent Non-Food
LNS	0.076^**^	0.075^**^	0.101^**^	0.111^**^	0.108^***^	0.113^***^	0.108^*^	0.112^*^
	(0.035)	(0.037)	(0.046)	(0.048)	(0.038)	(0.041)	(0.056)	(0.058)
11–18 mo postpartum					0.100^***^	0.109^***^	-0.108^***^	−0.183^***^
					(0.023)	(0.026)	(0.034)	(0.035)
LNS*11–18 mo postpartum					−0.069^*^	−0.080*	−0.014	−0.002
					(0.041)	(0.044)	(0.056)	(0.058)

N	1110	1133	1118	1097	1110	1133	1118	1097
Overall R^2^	0.166	0.139	0.356	0.386	0.168	0.141	0.356	0.386

Significance codes: ^***^(p < .01), ^**^(p < .05), ^*^(p < .1).

*Notes:* Expenditure data were collected from a random subsample of approximately 55% of households participating in the trial. Dependent variables are inverse hyperbolic sine, ln (yi + (yi^2^ + 1)^1/2^), of per capita total weekly food expenditures (columns 1 and 5), per capita weekly expenditures on nutrient-rich food groups (columns 2 and 6), per capita weekly expenditures on frequently purchased non-food items (columns 3 and 7), and per capita weekly expenditures on infrequently purchased non-food items (columns 4 and 8). Nutrient-rich food groups include animal-source foods, fruits, vegetables, pulses, and nuts. The variable ‘LNS’ is an indicator variable equal to one if the mother-infant pair was randomized to receive SQ-LNS and zero if the mother received IFA or MMN capsules and her infant received no supplementation. The variable ‘11–18 mo postpartum’ is an indicator variable for the interval (relative to the birth of the target infant) in which expenditure data were collected. The base interval is ‘5–11 mo postpartum’. Controls for interval of data collection, enumerator, year of maternal enrollment into the trial, maternal education, and household electrification are included in each model (unreported). Standard errors (in parentheses) are clustered at the household level. All regressions include a constant.

As shown in the first four columns of Table 3, over the course of the intervention, SQ-LNS provided to mothers during pregnancy and the first six months postpartum and to their infants from 6 to 18 months of age had a statistically significant and positive effect on per capita weekly total food expenditures, nutrient-rich food group expenditures, and frequent and infrequent non-food expenditures relative to households in which the mother received multiple micronutrient or iron-folic acid capsules.^22^ Given the inverse hyperbolic sine transformation of expenditures, the coefficient estimates imply that on average over the course of the intervention, relative to households in the non-LNS group, targeted SQ-LNS supplementation resulted in approximately 7.6% higher household per capita weekly food expenditures and 7.5% higher per capita weekly expenditures on nutrient-rich food groups, which include animal-source foods, fruits (excluding plantains), vegetables (excluding starchy vegetables such as cassava and yam), pulses, and nuts. It should be noted that reported food expenditures did not account for home-produced food, but given the semi-urban setting of the trial and the rarity of reported engagement in agriculture beyond small home gardens in the sample, the role of own-production is not likely to be influential. The targeted provision of SQ-LNS also increased per capita weekly expenditures on frequently purchased non-food goods and services by approximately 10% over the group who did not receive SQ-LNS. Similarly, expenditures on infrequently purchased goods and services were 11% higher in the LNS group.

The negative and statistically significant (p < .10) coefficients on the interaction between treatment group and the interval of data collection ‘11–18 mo postpartum’ in columns five and six of Table 3 suggest that the effect of the targeted provision of SQ-LNS to mothers and their infants on food and nutrient-dense food expenditures was larger during the interval from 5 to 11 months postpartum and less pronounced as the target child got older.

In the context of the randomized trial in Ghana, where households are largely food secure, dietary diversity is poor, micronutrient deficiencies are common, and overweight and obesity are increasing problems (Abrahams et al., 2011, World Bank, 2013), it is insightful to take a more disaggregated look at household food expenditures. Table 4 breaks down the effect of SQ-LNS on expenditures on each of the seven nutrient-rich food groups (top panel) and seven other food groups (bottom panel). The results show that targeted maternal and infant provision of SQ-LNS had a statistically significant positive effect on expenditures on fish, milk, and vegetables. Expenditures on some food groups not defined as nutrient-rich, including cereals, oils and fat, spices, sugar and sweets, and beverages were also statistically significantly higher among the LNS group.

**Table 4:** Effect of the provision of SQ-LNS to mothers and their infants on weekly expenditures by food groups.

		Meat	Poultry and Eggs	Fish	Milk	Fruit	Vegetables	Pulses and Nuts
Nutrient-Rich Food Groups	LNS	0.009	0.028	0.093^***^	0.049^***^	0.022	0.042^*^	0.014
		(0.027)	(0.027)	(0.032)	(0.018)	(0.018)	(0.024)	(0.013)
	N	1133	1133	1133	1133	1133	1133	1133
	R^2^ Overall	0.072	0.105	0.179	0.144	0.098	0.184	0.158

		Cereals	Oils and Fats	Starchy Staples	Spices	Sugar and Sweets	Beverages	Street Food

Other Food Groups	LNS	0.081^***^	0.024^*^	0.036	0.021^**^	0.026^**^	0.039^**^	−0.006
		(0.030)	(0.013)	(0.029)	(0.010)	(0.012)	(0.017)	(0.025)
	N	1126	1131	1130	1133	1132	1123	1133
	Overall R^2^	0.123	0.118	0.138	0.157	0.084	0.170	0.079

Significance codes: ^***^(p < .01), ^**^(p < .05), ^*^(p < .1).

*Notes:* Expenditure data were collected from a random subsample of approximately 60% of households participating in the trial. Dependent variables are inverse hyperbolic sine, ln (yi + (yi^2^ + 1)^1/2^), of weekly per capita expenditures in each food category. The variable ‘LNS’ is an indicator variable equal to one if the mother-infant pair was randomized to receive SQ-LNS and zero if the mother received IFA or MMN capsules and her infant received no supplementation. Controls for interval of data collection, enumerator, year of maternal enrollment into the trial, maternal education, and household electrification are included in each model (unreported). Standard errors (in parentheses) are clustered at the household level. All regressions include a constant.

### 5.2 Effect on labor income

Given higher expenditures in the LNS group, the next question logically centers on how households funded the relatively higher levels of expenditures. Since both food and non-food expenditures were higher in the LNS group, this suggests households were not simply reallocating their budget shares between food and non-food items. Regressions of expenditures on food as a percentage of total expenditures (unreported) confirm that there was no difference in the percentage of total expenditures allocated to food between households in the LNS and non-LNS groups. We therefore turn to an analysis of the effect of the targeted provision of SQ-LNS on labor income to explore whether a difference in income might have allowed for the relatively higher expenditures in the LNS group. In particular, we examine the effect of the intervention on the labor income of the target mother, her husband, and total per capita household labor income. For reference, in the non-LNS group over the course of the intervention, target mothers reported earning $9.03 (2011 USD) on average per week (standard deviation of $19.32), husbands earned $32.01 per week (standard deviation of $29.78), and average per capita weekly labor income for the household was $9.09 (standard deviation of $10.11).

The regression results in the first three columns of Table 5 show that over the course of the intervention, both per capita household labor income and paternal labor income were statistically significantly higher (p < .05) in the LNS group compared to the non-LNS group, while the targeted provision of SQ-LNS had no effect on the labor income of target mothers.^23^ The results indicate that per capita weekly household labor income was approximately 11% higher in LNS households on average over the course of the intervention, and among households in which the target mother had a husband, the income he earned from his primary work was approximately 11% higher. The husbands in the sample were primarily drivers, self-employed carpenters or masons, shop owners, and mechanics. Other household members were predominantly petty traders. Each of these jobs is conceivably flexible in terms of time spent working, and a likely source of relatively higher income working in these jobs is an adjustment in labor supply.

**Table 5:** Effect of the provision of SQ-LNS to mothers and their infants on weekly income.

	(1) Per Capita Household	(2) Target Mother	(3) Husband	(4) Per Capita Household	(5) Target Mother	(6) Husband
LNS	0.114^**^	0.027	0.109^**^	0.109^*^	−0.029	0.129^*^
	(0.049)	(0.080)	(0.055)	(0.063)	(0.099)	(0.071)
5–15 mo postpartum				−0.184^***^	−0.012	0.039
				(0.035)	(0.061)	(0.047)
15–22 mo postpartum				−0.045	0.653^***^	0.045
				(0.041)	(0.068)	(0.053)
LNS*5–15 mo postpartum				0.035	0.102	−0.029
				(0.059)	(0.100)	(0.083)
LNS*15–22 mo postpartum				−0.020	0.076	−0.036
				(0.069)	(0.112)	(0.089)

N	3208	3234	2170	3208	3234	2170
Overall R^2^	0.119	0.088	0.089	0.119	0.088	0.089

Significance codes: ^***^(p < .01), ^**^(p < .05), ^*^(p < .1).

*Notes:* Dependent variables are inverse hyperbolic sine, ln(yi+(yi^2^+1)^1/2^), of weekly: per capita household income (columns 1 and 4), income of target mother (columns 2 and 5), and income of target mother’s husband (columns 3 and 6). The variable ‘LNS’ is an indicator variable equal to one if the mother-infant pair was randomized to receive SQ-LNS and zero if the mother received IFA or MMN capsules and her infant received no supplementation. The variables ‘5–15 mo postpartum and ‘15–22 mo postpartum’ are indicator variables for the interval (measured relative to the birth of the target infant) in which income data were collected. The base interval is ‘late pregnancy – 5 mo postpartum’. Controls for interval of data collection, enumerator, year of maternal enrollment into the trial, maternal education, and household electrification are included in each model (unreported). Standard errors (in parentheses) are clustered at the household level. All regressions include a constant.

As with the expenditure data, we also explore heterogeneity in the effect of SQ-LNS on labor income by interval of data collection. The results, presented in columns 4–6 of Table 5, show that the effect of the targeted provision of SQ-LNS on each of the income variables did not vary across the three intervals in which income data were collected.

### 5.3 Spillover effect on sibling nutritional status

We have shown that food expenditures, including expenditures on nutrient-rich food groups, were higher in households in which the target mother and infant were provided with SQ-LNS relative to those in which the target mother received multiple micronutrient or iron-folic acid capsules. Estimates of the effect of the targeted intervention on expenditures for the subsample of households with a sibling under age five at maternal enrollment, shown in Table A15 of the Online Appendix, reveal a similar pattern of effects, though they are not as precisely estimated given the smaller sample size.

We now explore whether these observed differences in household behavior generated intrahousehold spillover effects on the nutritional status of the youngest sibling under age five in the household. The regression results presented in the first three columns of Table 6 show no spillover effects of the targeted intervention on the nutritional status (height-for-age z-score, weight-for-age z-score, and BMI-for-age z-score) of siblings, and the results in columns 4–6 show that this result holds at each interval that sibling measurements were taken.

**Table 6:** Effect of the provision of SQ-LNS to mothers and their infants on sibling anthropometric Z-scores.

	(1) HAZ	(2) WAZ	(3) BMIZ	(4) HAZ	(5) WAZ	(6) BMIZ
LNS	0.089	0.020	−0.075	0.102	0.014	−0.078
	(0.112)	(0.096)	(0.092)	(0.119)	(0.100)	(0.100)
15–22 mo postpartum				0.207^***^	−0.022	−0.224^***^
				(0.023)	(0.023)	(0.038)
LNS^*^15–22 mo postpartum				-0.026	0.012	0.005
				(0.050)	(0.046)	(0.073)

N	618	627	618	618	627	618
Overall R^2^	0.184	0.103	0.090	0.184	0.103	0.090

Significance codes: ^***^(p < .01), ^**^(p < .05), ^*^(p < .1).

*Notes:* Dependent variables are sibling height-for-age z-scores (columns 1 and 4), weight-for-age z-scores (columns 2 and 5), and BMI-for-age z-scores (columns 3 and 6). The variable ‘LNS’ is an indicator variable equal to one if the sibling’s mother and her infant were randomized to receive SQ-LNS and zero if the mother received IFA or MMN capsules and her infant received no supplementation. The variable ‘15–22 mo postpartum’ is indicator variables for the interval (measured relative to the birth of the target infant) in which sibling anthropometric measurements were taken. The base interval is ‘5–15 mo postpartum’. Controls for interval of data collection, anthropometrist, sibling age at enrollment, sibling gender, z-score of maternal height, maternal gestational age at enrollment, year of maternal enrollment into the trial, maternal education, and household electrification are included in each model (unreported). Standard errors (in parentheses) are clustered at the household level. All regressions include a constant.

We also explore potential sources of heterogeneity in the effect of SQ-LNS on sibling nutritional status by three factors: sibling age, sibling gender, and maternal height. Sibling age, gender, and baseline sibling z-scores were pre-specified as potential sources of heterogeneity in a statistical analysis plan. However, because baseline sibling z-scores were not collected for most siblings and because of the evidence linking improvements in linear growth with maternal height (described below), we opted to use maternal height in lieu of baseline z-scores after the analysis plan was posted online but before the analysis was conducted.

Several studies have shown an improvement in catch-up growth among children with taller mothers (examples include Adair, 1999, Crookston et al., 2010). These studies suggest the potential for a differential response in linear growth to improved nutrition by maternal height, and modification of the direct effect of SQ-LNS on stunting by maternal height among the target children in the households in this study (Adu-Afarwuah et al., 2016) adds further evidence of this possibility.

We find no statistically significant heterogeneity in sibling anthropometric status by age or gender. However, the regression results reported in Table 7 show a positive and significant (p < .05) coefficient on the interaction between the treatment indicator and the z-score of maternal height,^24^^,^^25^ suggesting that the targeted provision of maternal and infant SQ-LNS had a positive effect on the height-for-age z-score (HAZ) of siblings with relatively tall mothers.^26^

**Table 7:** Heterogeneity in sibling spillover effect on HAZ by Z-score of maternal height.

	(1) HAZ
LNS	0.065
	(0.110)
Z-Score of Maternal Height	0.315^***^
	(0.067)
LNS*Z- Score of Maternal Height	0.266^**^
	(0.116)

N	618
Overall R^2^	0.193

Significance codes: ^***^(p < .01), ^**^(p < .05), ^*^(p < .1).

*Notes:* Dependent variable is sibling height-for-age z-score. The variable ‘LNS’ is an indicator variable equal to one if the sibling’s mother and her infant were randomized to receive SQ-LNS and zero if the mother received IFA or MMN capsules and her infant received no supplementation. Controls for interval of data collection, anthropometrist, sibling age at enrollment, sibling gender, maternal gestational age at enrollment, year of maternal enrollment into the trial, maternal education, and household electrification are included in each model (unreported). Standard errors (in parentheses) are clustered at the household level. Regression model includes a constant.

**Fig. 2:**
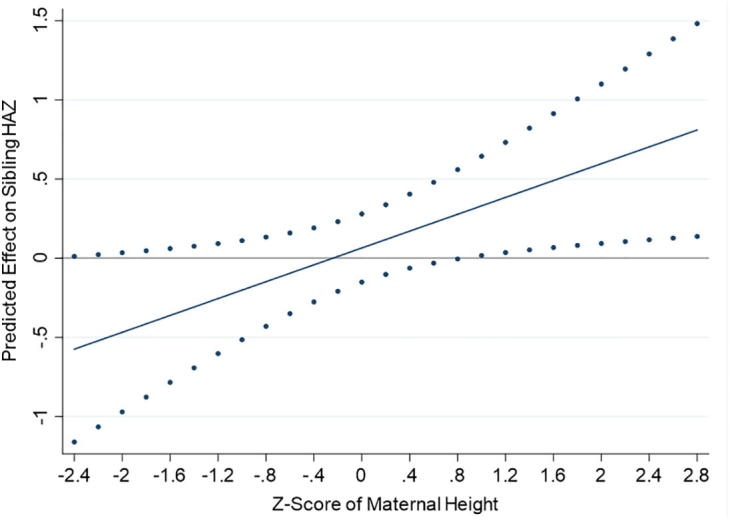
Effect of the provision of SQ-LNS to mothers and their infants on sibling HAZ by Z-score of maternal height with 95% confidence intervals.

Fig. 2 demonstrates the relationship graphically. For reference, among siblings in the non-SQ-LNS group, the average HAZ over the course of the trial was −1.18 (standard deviation of 1.12).

A child’s growth is a reflection of both genetic potential, which is revealed at least in part by maternal height (Addo et al., 2013), and environmental factors that influence growth, including nutrition and infection (Adair, 1999, Dewey and Mayers, 2011). At first measurement, the average HAZ of siblings with relatively taller mothers (z-score of height >0) was −1.01, which is statistically significantly higher (p < .01) than the average HAZ of −1.68 among siblings with relatively shorter mothers (z-score of height ≤0). Given this, the heterogeneity in the spillover effect on sibling HAZ by maternal height has several possible interpretations.

One interpretation is that, given their growth potential and relatively better HAZs early on in the trial, siblings with taller mothers were more responsive to higher consumption of nutrient-rich foods. This interpretation echoes Adair (1999) who found that among a population that was experiencing improvements in socioeconomic conditions including rising incomes and increased access to services like electricity and piped water, stunted children with taller mothers and those who were less severely stunted at baseline were more likely to exhibit catch-up growth. Another interpretation is that maternal height is a proxy for socioeconomic status, and better-off households, who were presumably less resource-constrained, had greater latitude to respond to the targeted intervention with higher food expenditures. Results reported in Table 8 generally show no heterogeneity in the treatment effect on food and nutrient-rich food group expenditures by maternal height, though column 4 of this table shows, for the sibling subsample, a marginally statistically significant larger effect of SQ-LNS on expenditures on nutrient-rich food groups in households with relatively taller mothers. Another possibility is that in better-off households, other features of the environment that may interact with nutrition to influence growth, such as water quality, sanitation, and hygiene practices, allowed for more responsiveness to the increase in food and nutrient-rich food group expenditures. Regressions (unreported) of a household sanitation index^27^ on maternal height, however, show no statistically significant association between the two in either the full sample or the sibling subsample.

**Table 8:** Heterogeneity in effect of the provision of SQ-LNS to mothers and their infants on food expenditures by Z-score of maternal height.

	Full Sample		Sibling Subsample		Non-Sibling Subsample	
	(1)	(2)	(3)	(4)	(5)	(6)
	Food	Nutrient-Rich Food Groups	Food	Nutrient-Rich Food Groups	Food	Nutrient-Rich Food Groups
LNS	0.068^*^	0.069^*^	0.070	0.059	0.060	0.068
	(0.036)	(0.038)	(0.058)	(0.066)	(0.046)	(0.047)
Z-Score of Maternal Height	−0.006	−0.004	−0.026	−0.040	0.016	0.023
	(0.019)	(0.019)	(0.036)	(0.039)	(0.022)	(0.022)
LNS^*^Z-Score of Maternal Height	0.031	0.035	0.119	0.170*	-0.013	-0.025
	(0.039)	(0.042)	(0.076)	(0.089)	(0.046)	(0.048)

N	1089	1111	394	403	695	708
Overall R^2^	0.094	0.089	0.165	0.144	0.062	0.065

Significance codes: ^***^(p < .01), ^**^(p < .05), ^*^(p < .1).

*Notes:* Dependent variables are inverse hyperbolic sine, ln (yi + (yi^2^ + 1)^1/2^), of per capita total weekly food expenditures (columns 1, 3, and 5) and of per capita weekly expenditures on nutrient-rich food groups (columns 2, 4, and 6). The variable ‘LNS’ is an indicator variable equal to one if the mother-infant pair was randomized to receive SQ-LNS and zero if the mother received IFA or MMN capsules and her infant received no supplementation. Nutrient-rich food groups include animal-source foods, fruits, vegetables, pulses, and nuts. Controls for interval of data collection, year of maternal enrollment into the trial, maternal education, and household electrification are included in each model (unreported). Standard errors (in parentheses) are clustered at the household level. Enumerator controls were omitted from these regressions because in the sibling subsample there was an enumerator control that was non-zero for only one cluster (household) such that there was not sufficient rank to perform the model test. All regressions include a constant.

## 6 Possible drivers of household behavioral responses

We have demonstrated higher household expenditures on both food and non-food items coupled with higher labor income in households in which the mother and her infant received SQ-LNS as well as some evidence of higher height-for-age z-scores among siblings with relatively tall mothers. There are several mechanisms or pathways through which households might have responded to the targeted intervention in ways that could have influenced expenditure patterns and/or labor income and, ultimately, the nutritional status of non-targeted household members. We describe them here and speculate on their likelihood, although the extent to which they can be empirically ruled out is limited by data availability.

The first potential mechanism is the direct sharing of SQ-LNS with the youngest sibling in the household. Although the women who participated in the randomized trial were explicitly instructed not to share their assigned supplement or the supplement provided to their infants, it is possible that supplements were shared with other household members. To assess the extent of sharing, during bi-weekly home visits to deliver supplements to mothers, home visitors on the iLiNS study staff asked the women about sharing of the supplement in the previous two weeks. Across all home visits throughout pregnancy and the first six months postpartum,^28^ 99.7% of the time women in SQ-LNS group reported sharing the supplement with no one, and just 0.25% of the time did they report sharing with a child. Among households with young children in the sibling subsample, reported rates of sharing were the same. Because these data were self-reported, their accuracy is uncertain and biased responses are possible given that women may have aimed to please project staff. So although we have no evidence supporting it, this mechanism cannot be entirely ruled out.

The second hypothesized mechanism is an income effect. If households were able to monetize SQ-LNS in an informal market, the money could have been used to fund additional consumption. And even if households were not selling SQ-LNS, households in which the mother and infant received SQ-LNS were different from those in which the mothers received capsules in that SQ-LNS contributed free calories to the household’s total food basket. This transfer of calories may have offset the household’s need to purchase those calories, thereby increasing the household budget. However, anecdotal evidence suggests that households were not selling SQ-LNS, and further, converting SQ-LNS to cash would have required substantial demand for the products. Given the complete novelty of SQ-LNS, it is unlikely private demand could have sustained the higher expenditures (Lybbert, Vosti, Adams, & Guissou, 2016). An income effect generated via the transfer of calories is also unlikely because the quantity of extra calories per day (118 kcal) was a very small percentage of a typical household’s total caloric needs.

The third potential mechanism is a freeing up of the household’s time constraint as a result of improvements in the health of mothers and infants consuming SQ-LNS that may have allowed for additional time spent working. That is, if mothers and infants who consumed SQ-LNS were generally less sick than those in the non-SQ-LNS group, this may have freed up additional household time, allowing for an increase in the household’s labor supply. The most common jobs reported in our sample – petty traders, drivers, artisans, shop owners, etc. – are, by and large, jobs that allow for flexibility in the time devoted to the job, so if SQ-LNS relaxed a household’s time constraint, it is conceivable that extra time could have been used working. Data on maternal and infant morbidity were also collected as part of the trial but have not yet been analyzed, and although the results of the analysis of those data may either lend credibility to or contradict this hypothesis, time-use data, which were not collected for the present study, would be necessary to legitimately test it.

While it is technically possible that the recommendation to mix SQ-LNS with food induced mothers to purchase more food, this is also unlikely in practice for several reasons. First, the quantity of SQ-LNS was small and mothers were free to mix it with whatever foods they wanted. Specifically, mothers were instructed to mix SQ-LNS for their own consumption with “one ladle of food (any food you want)”, and qualitative analysis of in-depth interviews showed that mothers typically mixed SQ-LNS with a range of commonly consumed foods, including porridge, soups, bread, tea, and stews (Klevor et al., 2016). For their infants, mothers were instructed to mix the SQ-LNS with “2–3 tablespoons of already prepared food”. It was also possible to consume SQ-LNS without mixing it with food, and in fact many mothers reported consuming SQ-LNS or feeding it to their infants without first mixing it with food. Finally, analysis of the infant and young child feeding practices among the study sample showed no difference in feeding practices when the target children were 18 months of age between those who received SQ-LNS and those who did not (Arimond et al., 2017).

Two final hypotheses are related to heightened maternal awareness of nutritious foods. The first hypothesis is that the way in which SQ-LNS were consumed relative to capsules had a priming effect on mothers. Priming is a theory of cognitive functioning in psychology used to describe an implicit memory process in which previous experience with a stimulus generates heightened sensitivity to a subsequent related stimulus (Henson, 2003, Schacter and Buckner, 1998). Mothers were advised to mix SQ-LNS with food and were told at the onset of the trial to eat foods like eggs, fruits, and vegetables whenever possible. The same message was conveyed again at 36 weeks of gestation and again when their infants began consuming SQ-LNS at six months. While mothers in the groups receiving capsules were also given the same information about feeding themselves and their infants nutritious foods, perhaps that act of fortifying food with SQ-LNS day-in and day-out had a priming effect on mothers in the SQ-LNS group, which made this message (and food in general) more salient and influenced the way mothers in the SQ-LNS group thought about the role of food in the production of health.

Experimental studies of the effect of priming on health behavior have found, for example, that priming can induce people to be more active (choose the stairs over an elevator) simply by being exposed to scrambled words related to being active (Wryobeck & Chen, 2003), or that priming people with the smell of a cleaning product induces them to keep their eating environment cleaner (Holland, Hendriks, & Aarts, 2005). In this intervention, the act of fortifying food with SQ-LNS every day may have had a priming effect that increased mothers’ sensitivity to food in general and/or increased the salience of the messages they received about the importance of consuming healthy foods. This heightened sensitivity may have influenced mothers’ decision-making surrounding food and motivated an increase in food consumption.

A second, related hypothesis is that mothers that received SQ-LNS were aware that the quality of their diet and the infant’s diet were improved because they were consuming SQ-LNS and sought to compensate other household members, who were not benefitting from the supplements, by providing more or different foods. Such a reaction might be explained by an aversion to unequal treatment of their children. Parental compensatory behavior like this has been observed in several settings, including Hsin (2012) in the United States, Adhvaryu and Nyshadham (2014) in Tanzania, Kazianga et al. (2014) in Burkina Faso, and Leight (2017) in China.

Since we found no effect of SQ-LNS on the labor income of target mothers, the plausibility of these last two hypotheses rests on mothers’ ability to spur changes in the labor supply of other household members in order to fund higher expenditures on food. Women who may have been in a good position in the household to spur such changes include more educated women, older women, and heads of household, all potential indicators of bargaining power within the household. Regressions of heterogeneity in the effect on household and paternal labor income show no difference in the effect by these maternal characteristics.

## 7 Limitations

Before discussing the implications of this collection of results, we consider limitations of the work. First, our study population is not a random sample of women in this area of Ghana, and certainly not of Ghana as a whole. Each woman in the study was actively seeking timely, formal prenatal care, and the characteristics and preferences of this sample of women, as well as the constraints they faced, were potentially different from those of women who do not seek out prenatal care. Similarly, households in the sample were, on average, food secure, and it is not clear whether this type of targeted intervention might elicit similar behavioral responses in less food secure settings.

Another consideration relates to the fact that the estimated effects of SQ-LNS on household behavior and sibling nutritional status were generated in the context of a randomized controlled trial in which SQ-LNS were delivered free of charge to households on a weekly or bi-weekly basis. In a more realistic distribution system, SQ-LNS would likely have higher time and, perhaps, financial costs that would be borne by the household (Lybbert, 2011). Depending on the specific mechanisms behind the observed behavioral responses, the way households responded to the intervention in the context of the randomized trial may not carry over when private costs of consuming SQ-LNS are higher. Related, the setting of this randomized trial, which was a busy commercial corridor with many self-employed people who, presumably, had the flexibility to adjust their labor supply, may have allowed households to respond to the intervention on margins that would not have been possible in settings in which the supply of labor was less flexible.

A final limitation of the study is that we are unable to address the intrahousehold distribution of food. While our results showed an increase in expenditures on food, including on nutrient-rich food groups, our data do not allow for an assessment of how the food was distributed within the household and thus we cannot determine the extent to which this behavioral response influenced the wellbeing of specific household members.

## 8 Conclusions

The results presented in this study show a behavioral response to a targeted maternal and infant nutrition intervention along dimensions outside the scope of the intervention. Our findings can be likened to local economy-wide effects of targeted transfers that provide cash, goods, and/or services to poor households. Evaluations of such transfers generally focus on impacts on the target households, but evidence has shown that given the interconnectedness (both socially and economically) of households within the local economy, targeted transfers can also generate spillover effects on non-target households (e.g., Filipski, Taylor, Thome, and Davis (2015)). This can occur when beneficiary households spend more money in the local economy, share resource with ineligible households, and/or model behavior change that is then adopted by ineligible households (Angelucci & Di Maro, 2016). In the case of within-household targeting, the interconnectedness of members within the micro-economy of the household means a transfer to one member can elicit behavioral responses that reverberate through the household and potentially affect the wellbeing of non-target members. We found that households in which the target mother and her infant received SQ-LNS, a food-based nutrient supplement designed to prevent undernutrition, had higher expenditures on food, including some nutrient-rich food groups, as well as higher expenditures on non-food goods and services. Household labor income was also higher, particularly among fathers, in households where the mother and her infant received SQ-LNS, suggesting a labor response. On average, the targeted intervention did not generate spillover effects on the nutritional status of non-targeted young siblings in the household. However, we found suggestive evidence of higher height-for-age z-scores in the LNS group compared to the non-LNS group among non-targeted children with mothers of relatively tall stature, which is an indicator of a child’s growth potential.

SQ-LNS is one tool available to help prevent undernutrition in mothers and young children, and if its consumption by these specific household members also brings about desirable changes in household food consumption patterns, the ‘value’ of SQ-LNS and programs that deliver it may be higher than what would be suggested based on maternal and child outcomes alone. Treated households in our sample did have higher expenditures on foods like sweets and beverages that may be considered undesirable from a nutritional perspective, but these households also spent more on nutrient-rich foods like fish, milk, and vegetables. Given that the nutrition messages delivered by the intervention were of very low intensity and that the limited information that was provided was the same across the treatment groups, these results suggest that something about preparing and consuming food with SQ-LNS every day – perhaps a physical effect or a psychological one – created circumstances in which households were willing to trade labor for additional consumption. If more could be learned about the specific mechanisms that were at work in driving these behavioral responses, policy makers might be able to leverage them in designing tools to promote healthy diets.

More broadly, the findings of this study point to the value in considering behavioral responses and spillovers in the design and in the analyses of targeted nutrition interventions. We showed that the seemingly benign introduction of a new food-based product into a household not only meant target household members began consuming that product, but it also triggered changes in household behavioral patterns with the potential to undermine or to enhance the intervention’s impact on the target household members and also to affect the wellbeing of non-target household members. Therefore, to *measure* the complete effects of such interventions, data on selected non-targeted household members may be required – data on the health, nutritional status, and development of non-targeted young siblings are likely priorities. To *understand* the effects of targeted interventions on household behavior, and to craft complementary policies (if needed), more and different types of data will be required. For example, dietary intake and anthropometric data over time for selected household members would provide a detailed look into intrahousehold food allocation, changes in food allocation patterns in response to an intervention, and the effects of these changes on food consumption and on nutritional status. *Ex ante* identification of other data that could be useful in measuring and understanding behavioral responses to targeted interventions is challenging, but data on food expenditures, income and income sources, and time use by care-givers and household heads are all likely candidates, and economic theory and the literature can expand the list for specific interventions. Armed with a deeper understanding of how and under what circumstances a targeted nutrition intervention might play out within the household, researchers can provide policy makers with a more comprehensive assessment of its expected associated costs and benefits, and suggestions regarding complementary policies if the evidence indicates the targeted intervention may be detrimental to the wellbeing of non-targeted household members.

## Footnotes

1 “Sibling” refers to the older brother or sister closest in age to the target infant participating in the randomized trial.

2 For more information on the iLiNS Project, see: http://ilins.org/.

3 Based on this recruitment mechanism, the women enrolled in the trial were not a random sample of pregnant women in this area of Ghana, which limits the generalizability of our results. We discuss this issue further when we address the limitation of the study.

4 Eligibility requirements were (1) at least 18 years of age, (2) not more than 20 weeks of gestation (determined by dating ultrasound), (3) possession of an antenatal card issued by the Ghana Health Service, (4) complete preliminary antenatal examination, (5) HIV negative or unknown status, (6) no chronic diseases requiring frequent medical attention, (7) residence in the Manya Krobo or Yilo Krobo districts throughout the intervention, and (8) prepared to sign an informed consent and receive home visitors. Women with known peanut or milk allergies, women participating in concurrent trials, and women with severe illnesses warranting hospital referrals were also excluded from the study.

5 Self-reported sharing of supplements was reported by mothers less than 1% of the time.

6 Exclusive breastfeeding was emphasized at all visits, and later visits also included information on appropriate complementary foods including the importance of dietary diversity and preparation techniques to minimize nutrient loss.

7 Take home rations of 10 kg of cereal flour were given to female students on a monthly basis conditional on a 90% school attendance rate.

8 Informal unions between the target mother and a partner were very common in the sample. These unions were informal in the sense that they had not received civil or traditional recognition, but for the purposes of this study we group men married to the target woman and men in an informal union with the target women together and call them husbands.

9 Households are excluded from analyses of paternal income in cases where the target mother did not have a husband or partner.

10 The z-score is calculated as the difference between the child’s value (weight or length) and the median value of the reference population divided by the standard deviation of the reference population, where the reference population is of the same gender and age.

11 The reference population is a sample of 8500 children from Brazil, Ghana, India, Norway, Oman, and the United States who were weighed and measured between 1997 and 2003 by the World Health Organization to generate growth curves based on a single international standard (WHO Multicentre Growth Reference Study Group, 2009).

12 As described below, the first round of sibling anthropometric measurements took place after maternal randomization for many, but not all, children. These first round measurements are therefore omitted from the present analysis but are summarized here to provide context.

13 A baseline round of data collection was also scheduled for each outcome variable, but because these data were collected pre-randomization for some households and post-randomization for other households, this first round of data collection was omitted from the analysis to avoid introducing bias.

14 In cases where no date of birth data were available for the target infant due to stillbirth, miscarriage, loss to follow-up at birth, etc., date of birth was estimated using the mother’s date of enrollment, her gestational age at enrollment, and the average gestational age at delivery in the sample.

15 As detailed in the Online Appendix, we do not have baseline measures of our outcome variables and other socioeconomic and demographic characteristics. Our assessment of balance at baseline is therefore limited to those variables captured at baseline.

16 The rate of permanent drop-out from the trial was very low (Adu-Afarwuah et al., 2015), while missed socioeconomic and sibling anthropometric measurement visits were more common due, primarily, to scheduling issues.

17 At the 5–11 month interval of household expenditure data collection, attrition was statistically significantly higher (p = 0.001) in the non-SQ-LNS group than the SQ-LNS group.

18 Note that we have defined the group of women who declined enrollment as those who made a choice not to enroll because, e.g., they did not wish to receive home visitors, their husband prohibited their enrollment, etc. This group does not include women who did not enroll due to reasons unrelated to choice, e.g., residence outside of the study area, >20 weeks of gestation, acute illness, etc.

19 One exception is that the estimated effect on frequently purchased non-food goods and services is no longer statistically significant (p = .12)

20 Clusters should be defined broadly enough to account for variation in both regressors and errors (Cameron & Miller, 2015). Given that the study site is relatively small and quite homogeneous, there does not exist a readily definable higher level of household groupings that could be used to define clusters.

21 The expenditure data are right-skewed, and we transform the data using the IHS function, defined as ln (y_i_ + (y_i_^2^ + 1)^1/2^) as shown in Burbidge et al., 1988, Pence, 2006. An alternative to the natural log, the IHS transformation is defined at zero and therefore allows us to include zeros without adding an arbitrary constant to expenditures. Except at very small values of y, the estimated coefficient with an IHS-transformed dependent variable can be interpreted as an approximate percentage change in y for a unit change in x (Pence, 2006).

22 This set of results is sensitive to controlling for enumerator. Without controlling for enumerator effects, the effect on nutrient-rich food groups is not statistically significant (p = .100), nor are the effects on frequently purchased non-food goods and services (p = .160) or infrequently purchased non-foods (p = .143).

23 Note that the results in Table 5 are based on the full sample of households who took part in the randomized trial. If we limit the sample to households for which expenditure data were collected (recall that this was a random subsample), the results, shown in Table A13 in the Online Appendix, also show a positive and statistically significant effect on household and paternal incomes.

24 Maternal height was standardized to make point estimates more interpretable.

25 A stratified analysis with the sample split between mothers with a positive z-score of height and those with a z-score at or below zero shows that the spillover effect of the targeted provision of maternal and infant SQ-LNS on sibling height-for-age z-scores is positive and statistically significant (p < .05) for siblings with mothers with positive z-scores while the effect is not statistically significant for siblings of mothers with z-scores at or below zero. These results are in Online Appendix Table A14.

26 Height-for-age is a measure of long-term nutritional status and is not sensitive to short-term fluctuations in nutrition. As such, we have excluded the first round of anthropometric measurements, which were taken within the first few months of randomization, since we would not expect an effect on height-for-age during that timeframe. We use the first round measurements as a placebo test to help confirm the estimated effects are attributable to the relationship between linear growth and maternal height. As expected, the results, available in Online Appendix Table A16, show no heterogeneity by maternal height when the analysis is limited to the first round of anthropometric measurements that occurred relatively shortly after maternal enrollment into the trial.

27 The household sanitation index was constructed using principal components analysis of primary water source in the dry and wet seasons, type of toilet, and visible condition of yard with respect to refuse.

28 Data on the self-reported extent of sharing of SQ-LNS over the course of the trial when the target infant was 6–18 months of age are not available.
