# Supplementary material for: Unintended effects of a targeted maternal and child nutrition intervention on household expenditures, labor income, and the nutritional status of non-targeted siblings in Ghana
Source: World Dev. 2018 Jul;107:138–50. doi: 10.1016/j.worlddev.2018.02.025 (PMC5917415; doi:10.1016/j.worlddev.2018.02.025)
Supplement: Supplementary data 1 [file mmc1.pdf]

Online Appendix

Unintended Effects of a Targeted Maternal and Child Nutrition Intervention on Household Expenditures, Labor Income, and the Nutritional Status of Non-Targeted Siblings in Ghana

A1: Construction of Outcome Variables

A1.1 Household Expenditures

Households were asked to recall their expenditures on an extensive set of food and non-food items, which were organized into categories (the household expenditure questionnaire is available upon request). Total food and non-food expenditures were then calculated as the sum over all relevant categories of expenditures. Sums were converted to USD using the average monthly exchange rate and adjusted to 4th quarter 2011 USD. Per capita[1] household food expenditures were calculated by dividing total food expenditures by the number of household members reported as eating at least eight of their meals with the household during the previous seven days. Per capita short- and long-term non-food expenditures were calculated by dividing total short- and total long-term expenditures by the household size (that is, all permanent household members regardless of whether they consumed most of their meals with the household in the week prior to the interview).

A1.2 Household Income

Labor income for each household member was collected as part of the demographic and socioeconomic characteristics questionnaire (available upon request). The questionnaire respondent, who was the target mother enrolled in the randomized trial, was asked to report the income each household member typically received from their primary work. The income of each household member was converted to a weekly rate and converted to USD using the average monthly exchange rate and adjusted to 4th quarter 2011 USD. Per capita total household income was calculated as the sum of each household member's income divided by the household size.

A1.3 Sibling Anthropometrics

For our analysis we constructed z-scores of the sibling anthropometric measures, which enables the comparison of an individual child's anthropometric measurements (length/height and weight) to children in the reference population (O'Donnell et al., 2008). The z-score is calculated as the difference between the child's value (weight or height) and the median value of the reference population divided by the standard deviation of the reference population, where the reference population is of the same gender and age. The reference population is a sample of 8,500 children from Brazil, Ghana, India, Norway, Oman, and the United States who were weighed and measured between 1997 and 2003 by the World Health Organization to generate growth curves based on a single international standard (WHO Multicentre Growth Reference Study Group, 2009).

[1] Expenditures per adult equivalent rather than per capita may be a better representation of how much a household spent relative to other households with different demographic compositions. Missing age data and complications in the way the household roster was updated between rounds prevent us from calculating accurate per adult equivalents in this case.

We calculated z-scores of height-for-age (HAZ), weight-for-age (WAZ) and BMI-for-age (BMIZ) using WHO Anthro and WHO2007, Stata macros from the World Health Organization based on the updated WHO child growth standards and WHO reference 2007, respectively. WHO Anthro calculates z-scores for children under 60 months, and WHO2007 calculates z-scores for children 60 months and older. Since some siblings in the sample aged past 60 months during the intervention, both macros were needed to generate z-scores for these children across all three rounds. Available for download at: http://www.who.int/childgrowth/software/en/ (WHO Anthro) and http://www.who.int/growthref/tools/en/ (WHO2007). Because these macros do not calculate weight-for-height z-scores (WHZ) for children over 60 months (and therefore WHZ is missing for children who aged past 60 months at follow-up anthropometric visits), WHZ was not included in this analysis. In four cases, siblings were recorded as losing height between rounds; these children were excluded from analyses of HAZ and BMIZ. An additional three biologically implausible z-scores were also excluded. Biologically implausible z-scores are outside the range -6 to 6 for HAZ, -6 to 5 for WAZ, and -5 to 5 for BMIZ (Mei and Grummer-Strawn, 2007) and can be attributed to improper measurement or data entry error.

A2. Delays in Timing of Enumeration

The timing of enumeration in our data warrants special consideration because it raises concerns about our ability to use the data to draw valid causal inferences. In particular, each questionnaire was scheduled to be administered at either two or three specific time points for follow-up during the trial. However, scheduling issues, primarily,[2] meant socioeconomic questionnaires and sibling anthropometric measurements were often administered weeks or months after they were scheduled, and some visits were missed entirely. Therefore, we have a short, unbalanced panel of observations for each household or sibling, and the timing of each follow-up observation relative to, e.g., the birth of the target infant, varies substantially in the data.

It is possible that delays in enumeration were endogenous if, for example, home visits to administer questionnaires were more likely to be rescheduled/delayed for some particularly entrepreneurial households who were often away from home working, in which cases delays in enumeration were at least partly attributed to the household's entrepreneurial spirit, which is also likely associated with our outcomes of interest and may be influenced by the treatment. If delays in enumeration were affected by the treatment, including it as a control in our regression models could bias the estimated effects by capturing some of the impact (Duflo, Glennerster, and Kremer, 2008). Although (unreported) regressions of the delay in enumeration on the treatment indicator variable indicate that, for each outcome variable, variation in the timing of enumeration was balanced between treatment groups, this does not ensure the delays were random. We address this concern with a robustness check for our main results, described next.

To confirm that our estimated treatment effects do not change if we control for the (possibly endogenous) delay in enumeration (i.e., the number of months from the scheduled to actual enumeration of a specific questionnaire), we instrument for this delay with several instruments. Our first instrument is the duration of time from the beginning of the trial to maternal enrollment into the trial. Given the rolling enrollment trial design (enrollment took place over a two year period from

[2] Scheduling issues included difficulty scheduling a time to administer lengthy socioeconomic questionnaires, frequent rescheduling of visits when respondents were not home/available, and scheduling conflicts with the anthropometry team in the case of the sibling anthropometric measurements.

December 2009 – December 2011), the iLiNS socioeconomic team had a lot of time to become familiar with the study area, improve they interview scheduling system, and hone their tactics for tracking down survey respondents for interviews. As such, the duration in time from the start of the trial in December 2009 to maternal enrollment negatively correlated with delays in enumeration but is uncorrelated with the error term in our estimating equations.

Our second instrument is the number of households enrolled in the trial per enumerator at the time of scheduled enumeration. Again given the rolling enrollment design of the intervention, there were few households enrolled in the trial early on, and the ratio of households to enumerators was low. As more households were enrolled, additional enumerators were hired on, but not at a rate to maintain the households per enumerator ratio, so this ratio also increased. Towards the end of the trial as households were completing the intervention but no new households were being enrolled, the ratio again decreased. This provides an instrument that is positively correlated with delays in but is uncorrelated with the error in our estimate equations.

In tables A5-A8 below, we present our main results estimated without controlling for the delay in enumeration side-by-side with the results when controlling for the delay (where we instrument for the delay using the instruments described above).

Table A5 shows this set of results for household expenditures. Based on the Kleibergen-Paap F-statistics presented in columns 2, 4, and 6, we reject the null of weak identification using the ‘months from start of trial’ and ‘households per enumerator’ instruments (Stock and Yogo, 2005).[3] The random effects two-stage least squares (2SLS) coefficient estimates on the treatment variables are also very close to the estimates obtained by directly control for timing of enumeration.

Table A6 similarly shows the estimated effects on income are robust controlling for delays in enumeration of the income questionnaire, thought the estimated effect of randomization into the LNS group on household income is smaller in magnitude (and statistically significant at the 10% rather than the 5% level) when we control for the delay in enumeration.

The estimated effects on sibling anthropometrics, shown in Table A7, also confirm these results are not sensitive to controlling for delays in sibling measurements. And finally, the interactions between LNS and z-score of maternal height, shown in Table A8, confirm that the evidence of a positive spillover effect of LNS on sibling HAZ among siblings with taller mothers is robust to controlling for delays in sibling measurements (though the statistical significance of the interaction falls from the 5% to the 10% level).

Together, this set of robustness checks confirms that accounting for the delays in enumeration do no change any of our main results in any significant ways.

[3] The critical values suggested by Stockand Yogo (2005) are calculated for the Cragg-Donald F statistic and are appropriate under i.i.d. errors. Staigerand Stock (1997) suggest caution with applying these critical values to the Kleibergen-Paap F statistic in the presence of non-i.i.d. errors or to alternatively apply the rule of thumb that the F statistic should be greater than 10 to reject the null of weak identification.

A3. Supplementary Tables

Table A1. Statements read to women upon enrollment into the randomized trial

To women receiving **Capsules**:

1. The capsules are all for you because women need more vitamins and minerals when they are pregnant.
2. You will have to take one capsule per day, every day in the week.
3. Do not share the capsules with others.
4. You will take the capsule with water in the morning after you have eaten. If you forget to take it in the morning, then take it in the afternoon after you have eaten. If you forget it in the morning and also in the afternoon, then take it in the evening after you have eaten. If you forget to take the capsule during the whole day, do not take two capsules the next day; it is always one capsule per day.
5. If you need to travel for a number of days, take the capsules with you so you can take them every day, whilst you are away.
6. Do not forget to eat meat, fish, eggs, fruits and vegetables whenever you can. You still need these foods even if you take the capsules we have given you.

To women receiving **LNS-P&L**:

1. This *Nkatepa** supplement is all for you because women need more vitamins and minerals when they are pregnant.
2. You will have to eat one sachet per day, every day in the week.
3. Do not share the *Nkatepa* with others.
4. Mix the entire content of one sachet of Nkatepa with one ladle of food (any food you want) in the morning and eat. The one ladle of food is to make sure that you eat the amount of supplement you need for the day and not leave some of it mixed with food behind and let it go to waste. When that happens, it means you did not eat the amount of supplement your body needs for the day.
5. After you have eaten the one ladle of food mixed with the supplement, you can then go ahead and eat more of your food.
6. If you forget to eat the supplement in the morning, then eat it in the afternoon. If you forget it in the morning and also in the afternoon, then eat it in the evening.
7. If you forget the supplement the whole day, do not eat two sachets the next day; it is always one sachet per day.
8. If you need to travel for a number of days, take the *Nkatepa* with you so you can take them every day, whilst you are away.
9. Do not forget to eat meat, fish, eggs, fruits and vegetables whenever you can. You still need these foods even if you eat the *Nkatepa* we have given you.

*Note Nkatepa is the local name for LNS.

Table A2. Statements read to women when their infants were 6 months

To women whose infants are receiving **no supplementation**:
1. Breastfeed your baby as you did before.
2. Please do not forget to give your baby other things such as eggs, fruits and vegetables whenever you can. Your baby still needs these foods.

To women whose infants are receiving **Nkatepa**:
1. Breastfeed your baby as you did before.
2. This Nkpatepa* supplement is for your baby because babies need special foods from 6 months of age; do not share it with others.
3. The baby will need to eat two (2) sachets per day, every day in the week. That is, you will give one sachet in the morning and another sachet in the afternoon or evening.
4. Each time you are giving the supplement to your baby, here is what you will do:
   - You will mix the entire content of the sachet with 2-3 tablespoons of already prepared food and feed it to the baby.
   - After the baby has eaten the 2-3 tablespoons mixed with the supplement, you can go ahead and give him/her more of the food. The 2-3 tablespoons is to make sure that the baby eats all of the supplement in the sachet and not leave some of it mixed with food behind and let it go to waste.
   - You can mix the Nkatepa with any food you are giving to the baby.
5. Do not cook food with the supplement; store the supplement at room temperature; you do not need to keep the supplement in the refrigerator.
6. If one day you did not give the supplement to your baby at all, or gave only one sachet instead of two sachets, do not give more than two sachets the next day; it is always two sachets per day.
7. In case you need to travel with the baby for a number of days, take the Nkatepa with you so you can give it to the baby every day, whilst you are away.
8. Do not forget to give your baby other things such as eggs, fruits and vegetables whenever you can. You baby still needs these foods even if you give him/her *Nkatepa*

*Note Nkatepa is the local name for LNS.

Table A3. Balance in Background Characteristics by Interval of Data Collection

		Late Pregnancy – 5mo		5-11 mo[a] | 5-15 mo[b]		11-18 mo[a] | 15-22mo[b]	
	Variable	N	Mean	N	Mean	N	Mean
Panel A: Income	Maternal Age	1,179	26.78	1,058	26.91	997	26.75
	Maternal Education	1,179	7.43	1,058	7.43	997	7.48
	Children	1,179	1.18	1,058	1.20	997	1.19
	Maternal Height	1,161	158.89	1,040	158.91	985	158.93
	Maternal Gestational Age	1,179	16.20	1,058	16.19	997	16.19
	Maternal Supplement Use	1,179	0.89**	1,058	0.88*	997	0.88*
	Electricity	1,179	0.85	1,058	0.85	997	0.85
Panel B: Household Expenditures	Maternal Age			588	27.02	545	26.89
	Maternal Education			588	7.29	545	7.35
	Children			588	1.25	545	1.23
	Maternal Height			577	158.68	534	158.71
	Maternal Gestational Age			588	16.29	545	16.27
	Maternal Supplement Use			588	0.88	545	0.88
	Electricity			588	0.86	545	0.85
Panel C: Sibling Anthropometry	Maternal Age			321	28.32	306	28.16
	Maternal Education			321	7.25	306	7.31
	Children			321	1.92	306	1.89
	Maternal Height			321	159.38	306	158.92
	Maternal Gestational Age			321	16.43	306	16.34
	Maternal Supplement Use			321	0.87	306	0.88
	Electricity			321	0.83	306	0.84
	Sibling Age			321	35.20	306	35.56
	Sibling Female			321	0.51	306	0.51

[a]Relevant intervals for household expenditures data collection. [b]Relevant intervals for income and sibling anthropometric data collection.

Significance codes: *** (p < .01), ** (p < .05), * (p < .1) indicate difference in means between SQ-LNS group and non-SQ-LNS group.

Notes: Income data were collected from the full sample of households. Expenditure data were collected from a random subsample of households. The sample size for 'sibling' variables is limited to households with a sibling who was under age five at maternal enrollment into the trial.

Table A4. Selection

Variable	Definition	Enrolled N	Enrolled Mean	Enrolled Std. Deviation	Eligible but Refused Enrollment N	Eligible but Refused Enrollment Mean	Eligible but Refused Enrollment Std. Deviation	P-Value*
Maternal Age	Maternal age in years	1,320	26.71	5.52	146	28.19	5.97	0.002
Primary Education	= 1 if target mother has at least primary level of education	1,320	0.78	0.41	146	0.76	0.43	0.53
Children	Number of target mother's living biological children	1,320	1.17	1.23	146	1.22	1.28	0.65
Mobile Phone	= 1 if mother owns a mobile phone	1,320	0.70	0.46	146	0.73	0.44	0.46
Krobo	= 1 if mother's native language is Krobo	1,320	0.72	0.45	146	0.66	0.48	0.12

*P-value for two-tailed t-test of difference in means between mothers who were enrolled and those who were eligible for enrollment but did not enroll.

Table A5. Effect of SQ-LNS on Per Capita Daily Household Expenditures

	(1) Food	(2) Food	(3) Nutrient-Rich Food	(4) Nutrient-Rich Food	(5) Frequent Non-Food	(6) Frequent Non-Food	(7) Infrequent Non-Food	(8) Infrequent Non-Food
LNS	0.076**	0.075**	0.075**	0.075**	0.101**	0.102**	0.111**	0.113**
	(0.035)	(0.035)	(0.037)	(0.037)	(0.046)	(0.045)	(0.048)	(0.048)
IV for Delay	NO	YES	NO	YES	NO	YES	NO	YES
N	1110	1110	1133	1133	1118	1118	1097	1097
Overall R^2	0.166	0.165	0.139	0.139	0.356	0.355	0.386	0.368
Kleibergen-Paap F-Stat		28.383		30.095		30.420		29.815

Significance codes: *** (p < .01), ** (p < .05), * (p < .1).

Notes: Dependent variables are inverse hyperbolic sine, $\ln(yi+(yi^2+1)^{1/2})$, of per capita weekly food expenditures (columns 1 and 2), per capita weekly expenditures on nutrient-rich food groups for (columns 3 and 4), per capita weekly expenditures on frequently purchased non-food items (columns 5 and 6), and per capita weekly expenditures on infrequently purchased non-food items for (columns 7 and 8). Nutrient-rich food groups include animal-source foods, fruits, vegetables, pulses, and nuts. The variable 'LNS' is an indicator variable equal to one if the mother-infant pair was randomized to receive SQ-LNS and zero if the mother received IFA or MMN capsules and her infant received no supplementation. Controls for interval of data collection, enumerator, year of maternal enrollment into the trial, maternal education, and household electrification are included in each model (unreported). 'IV for Delay' indicates 2SLS was used to instrument for the delay in enumeration (months from scheduled to actual date of enumeration) with the instruments 'households per enumerator' and 'months from start of trial'. Standard errors (in parentheses) are clustered at the household level. All regressions include a constant.

Table A6. Effect of SQ-LNS on Income

	(1) Per Capita Household	(2) Per Capita Household	(3) Target Mother	(4) Target Mother	(5) Paternal	(6) Paternal
LNS	0.114**	0.053*	0.027	0.021	0.109**	0.093**
	(0.049)	(0.029)	(0.080)	(0.041)	(0.055)	(0.042)
IV for Delay	NO	YES	NO	YES	NO	YES
N	3208	3208	3234	3234	2170	2170
Overall R^2	0.119	0.148	0.088	0.099	0.089	0.127
Kleibergen-Paap F-Stat		53.086		53.814		38.742

Significance codes: *** (p < .01), ** (p < .05), * (p < .1).

Notes: Dependent variables are inverse hyperbolic sine, $\ln(yi+(yi^2+1)^{1/2})$, of weekly per capita household income (columns 1 and 2), weekly income of target mother (columns 3 and 4), and weekly income of target mother's husband (columns 5 and 6). The variable 'LNS' is an indicator variable equal to one if the mother-infant pair was randomized to receive SQ-LNS and zero if the mother received IFA or MMN capsules and her infant received no supplementation. Controls for interval of data collection, enumerator, year of maternal enrollment into the trial, maternal education, and household electrification are included in each model (unreported). 'IV for Delay' indicates 2SLS was used to instrument for the delay in enumeration (months from scheduled to actual date of enumeration) with the instruments 'households per enumerator' and 'months from start of trial'. Standard errors (in parentheses) are clustered at the household level. All regressions include a constant.

Table A7. Effect of SQ-LNS on Sibling Anthropometric Z-Scores

	(1) HAZ	(2) HAZ	(3) WAZ	(4) WAZ	(5) BMIZ	(6) BMIZ
LNS	0.089	0.050	0.020	-0.008	-0.075	-0.095
	(0.112)	(0.127)	(0.096)	(0.101)	(0.092)	(0.093)
IV for Delay	NO	YES	NO	YES	NO	YES
N	618	618	627	627	618	618
Overall R^2	0.184	0.054	0.103	0.048	0.090	0.102
Kleibergen-Paap F-Stat		29.093		29.090		29.553

Significance codes: *** (p < .01), ** (p < .05), * (p < .1).

Notes: Dependent variables are sibling height-for-age z-scores (columns 1 and 2), weight-for-age z-scores (columns 3 and 4), and BMI-for-age z-scores (columns 5 and 6). The variable 'LNS' is an indicator variable equal to one if the sibling's mother and her infant were randomized to receive SQ-LNS and zero if the mother received IFA or MMN capsules and her infant received no supplementation. Controls for interval of data collection, anthropometrist, sibling age at enrollment, sibling gender, z-score of maternal height, maternal gestational age at enrollment, year of maternal enrollment into the trial, maternal education, and household electrification are included in each model (unreported). 'IV for Delay' indicates 2SLS was used to instrument for the delay in enumeration (months from scheduled to actual date of enumeration) with the instruments 'households per enumerator', 'months from start of trial', and 'socioeconomic enumerator'. Standard errors (in parentheses) are clustered at the household level. All regressions include a constant.

Table A8. Heterogeneity in Sibling Spillover Effect on HAZ by Z-Score of Maternal Height

	(1)<br>HAZ	(2)<br>HAZ
LNS	0.065	0.035
	(0.110)	(0.117)
Z-Score of Maternal Height	0.315***	0.337***
	(0.067)	(0.070)
LNS*Z-Score of Maternal Height	0.266**	0.233*
	(0.116)	(0.122)
IV for Delay	NO	YES
N	618	618
Overall R^2	0.193	0.103
Kleibergen-Paap F-Stat		28.848

Significance codes: *** (p < .01), ** (p < .05), * (p < .1).

Notes: Dependent variables are sibling height-for-age z-scores. The variable 'LNS' is an indicator variable equal to one if the sibling's mother and her infant were randomized to receive SQ-LNS and zero if the mother received IFA or MMN capsules and her infant received no supplementation. Controls for interval of data collection, anthropometrist, sibling age at enrollment, sibling gender, z-score of maternal height, maternal gestational age at enrollment, year of maternal enrollment into the trial, maternal education, and household electrification are included in each model (unreported). 'IV for Delay' indicates 2SLS was used to instrument for the delay in enumeration (months from scheduled to actual date of enumeration) with the instruments 'households per enumerator', 'months from start of trial', and 'socioeconomic enumerator'. Standard errors (in parentheses) are clustered at the household level. All regressions include a constant.

Table A9. Effect of SQ-LNS and MMN on Per Capita Daily Household Expenditures

	(1)<br>Food	(2)<br>Nutrient-Rich Food	(3)<br>Frequent Non-Food	(4)<br>Infrequent Non-Food
LNS	0.081**	0.080*	0.086	0.098*
	(0.040)	(0.043)	(0.055)	(0.055)
MMN	0.010	0.009	-0.031	-0.027
	(0.037)	(0.039)	(0.053)	(0.054)
N	1110	1133	1118	1097
Overall R^2	0.166	0.139	0.356	0.386s

Significance codes: *** (p < .01), ** (p < .05), * (p < .1).
Notes: LNS refers to the mother/infant dyad randomized to the SQ-LNS group, and MMN refers to the mother/infant dyad randomized to the multiple micronutrient supplement group. The omitted group is iron-folic acid. Dependent variables are inverse hyperbolic sine, ln(yi+(yi2+1)1/2), of per capita total weekly food expenditures (1), per capita weekly expenditures on nutrient-rich food groups (2), per capita weekly expenditures on frequently purchased non-food items (3), and per capita weekly expenditures on infrequently purchased non-food items (4). Nutrient-rich food groups include animal-source foods, fruits, vegetables, pulses, and nuts. Controls for interval of data collection, enumerator, year of maternal enrollment into the trial, maternal education, and household electrification are included in each model (unreported). Standard errors (in parentheses) are clustered at the household level. All regressions include a constant.

Table A10. Effect of SQ-LNS and MMN on Daily Income

	(1)<br>Per Capita Household	(2)<br>Target Mother	(3)<br>Husband
LNS	0.099*	0.007	0.150**
	(0.057)	(0.091)	(0.067)
MMN	-0.030	-0.039	0.082
	(0.060)	(0.091)	(0.068)
N	3208	3234	2170
Overall R^2	0.119	0.088	0.090

Significance codes: *** (p < .01), ** (p < .05), * (p < .1).
Notes: LNS refers to the mother/infant dyad randomized to the SQ-LNS group, and MMN refers to the mother/infant dyad randomized to the multiple micronutrient supplement group. The omitted group is iron-folic acid. Dependent variables are inverse hyperbolic sine, $\ln(yi+(yi^2+1)^{1/2})$, of weekly: (1) per capita household income, (2) income of target mother, and (3) income of target mother's husband. Controls for interval of data collection, enumerator, year of maternal enrollment into the trial, maternal education, and household electrification are included in each model (unreported). Standard errors (in parentheses) are clustered at the household level. All regressions include a constant.

Table A11. Effect of SQ-LNS and MMN on Sibling Anthropometric Z-Scores

	(1) HAZ	(2) WAZ	(3) BMIZ
LNS	0.110	0.071	-0.008
	(0.124)	(0.110)	(0.107)
MMN	0.042	0.100	0.131
	(0.129)	(0.115)	(0.107)
N	618	627	618
Overall R^2	0.184	0.105	0.092

Significance codes: *** (p < .01), ** (p < .05), * (p < .1).
Notes: LNS refers to the mother/infant dyad randomized to the SQ-LNS group, and MMN refers to the mother/infant dyad randomized to the multiple micronutrient supplement group. The omitted group is iron-folic acid. Dependent variables are (1) sibling height-for-age z-scores, (2) weight-for-age z-scores, and (3) BMI-for-age z-scores. Controls for interval of data collection, anthropometrist, sibling age at enrollment, sibling gender, z-score of maternal height, maternal gestational age at enrollment, year of maternal enrollment into the trial, maternal education, and household electrification are included in each model (unreported). Standard errors (in parentheses) are clustered at the household level. All regressions include a constant.

Table A12. Heterogeneity in Sibling Spillover Effect on HAZ by Z-Score of Maternal Height

	(1) HAZ
LNS	0.087
	(0.123)
Maternal Height	0.294***
	(0.083)
LNS*Maternal Height	0.287**
	(0.129)
MMN	0.045
	(0.128)
MMN*Maternal Height	0.037
	(0.131)
N	618
Overall R^2	0.193

Significance codes: *** (p < .01), ** (p < .05), * (p < .1).
Notes: LNS refers to the mother/infant dyad randomized to the SQ-LNS group, and MMN refers to the mother/infant dyad randomized to the multiple micronutrient supplement group. The omitted group is iron-folic acid. Dependent variable is sibling height-for-age z-score. Controls for interval of data collection, anthropometrist, sibling age at enrollment, sibling gender, z-score of maternal height, maternal gestational age at enrollment, year of maternal enrollment into the trial, maternal education, and household electrification are included in each model (unreported). Standard errors (in parentheses) are clustered at the household level. All regressions include a constant.

Table A13. Effect on Income - Expenditure Subsample

	(1)<br>Per Capita Household	(2)<br>Target Mother	(3)<br>Husband
LNS	0.096**	0.062	0.112**
	(0.037)	(0.056)	(0.055)
N	1823	1841	1258
Overall R^2	0.140	0.101	0.129

Significance codes: *** (p < .01), ** (p < .05), * (p < .1).

Notes: Dependent variables are inverse hyperbolic sine, $\log(yi+(yi^2+1)^{1/2})$, of daily (1) per capita household income, (2) income of target mother, and (3) income of target mother's husband. The variable 'LNS' is an indicator variable equal to one if the mother-infant pair was randomized to receive SQ-LNS and zero if the mother received IFA or MMN capsules and her infant received no supplementation. Controls for timing of enumeration relative to the birth of the target infant, enumerator, season and year of maternal enrollment into the trial, language primarily spoken at home, and maternal gestational age at enrollment, z-score of height, and education are included in the model (unreported). Standard errors (in parentheses) are clustered at the household level. All regressions include a constant.

Table A14. Stratified Analysis of Sibling Spillover Effect on HAZ by Maternal Height

	(1)<br>Z-Score of Maternal<br>Height > 0	(2)<br>Z-Score of Maternal<br>Height <= 0
LNS	0.344**	-0.139
	(0.159)	(0.150)
N	309	309
Overall R^2	0.164	0.112

Significance codes: *** (p < .01), ** (p < .05), * (p < .1).

Notes: Analysis is stratified by z-score of maternal height. Dependent variable is sibling height-for-age z-score. The variable 'LNS' is an indicator variable equal to one if the mother-infant pair was randomized to receive SQ-LNS and zero if the mother received IFA or MMN capsules and her infant received no supplementation. Controls for interval of data collection, anthropometrist, sibling age at enrollment, sibling gender, z-score of maternal height, maternal gestational age at enrollment, year of maternal enrollment into the trial, maternal education, and household electrification are included in each model (unreported). Standard errors (in parentheses) are clustered at the household level. All regressions include a constant.

Table A15. Effect on Per Capita Weekly Household Expenditures - Sibling Subsample

	(1)<br>Food	(2)<br>Nutrient-Rich Food Groups	(3)<br>Frequent Non-Food	(4)<br>Infrequent Non-Food
LNS	0.079	0.072	0.132	0.141
	(0.059)	(0.067)	(0.105)	(0.115)
N	394	403	388	385
Overall R^2	0.154	0.126	0.157	0.150

Significance codes: *** (p < .01), ** (p < .05), * (p < .1).

Notes: Dependent variables are inverse hyperbolic sine, $\ln(yi+(yi^2+1)^{1/2})$, of (1) per capita total weekly food expenditures, (2) per capita weekly expenditures on nutrient-rich food groups, (3) per capita weekly expenditures on frequently purchased non-food items, and (4) per capita weekly expenditures on infrequently purchased non-food items. Nutrient-rich food groups include animal-source foods, fruits, vegetables, pulses, and nuts. The variable 'LNS' is an indicator variable equal to one if the mother-infant pair was randomized to receive SQ-LNS and zero if the mother received IFA or MMN capsules and her infant received no supplementation. Controls for interval of data collection, year of maternal enrollment into the trial, maternal education, and household electrification are included in each model (unreported). Standard errors (in parentheses) are clustered at the household level. Enumerator controls were omitted from these regressions because in the sibling subsample there was an enumerator control that was non-zero for only one cluster (household) such that there was not sufficient rank to perform the model test. All regressions include a constant.

Table A16. Short-Term Heterogeneity in Sibling Spillover Effect on HAZ by Z-Score of Maternal Height

	(1)<br>HAZ
LNS	0.010
	(0.125)
Z- Score of Maternal Height	0.407***
	(0.074)
LNS X Z- Score of Maternal Height	0.126
	(0.132)
N	410
R^2	0.174
Prob > F	0.000

Significance codes: *** (p < .01), ** (p < .05), * (p < .1).

Notes: Dependent variable is the first round of sibling height-for-age z-score. The variable 'LNS' is an indicator variable equal to one if the sibling's mother and her infant weres randomized to receive SQ-LNS and zero if the mother received IFA or MMN capsules and her infant received no supplementation. Controls for interval of data collection, anthropometrist, sibling age at enrollment, sibling gender, maternal gestational age at enrollment, year of maternal enrollment into the trial, maternal education, and household electrification are included in each model (unreported). Robust standard errors in parentheses. All regressions include a constant.

References

Duflo, Esther, Rachel Glennerster, and Michael Kremer. 2008. Using Randomization in Development Economics Research: A Toolkit. In *T. Schultz and John Strauss, eds., Handbook of Development Economics* (Vol. 4). Amsterdam and New York: North Holland.

Mei, Zuguo, and Laurence Grummer-Strawn. 2007. "Standard Deviation of Anthropometric Z-scores as a Data Quality Assessment Tool Using the 2006 WHO Growth Gtandards: A Cross Country Analysis." *Bulletin of The World Health Organization* 85, no. 6: 441-448.

O'Donnell, Owen, Eddy van Doorslaer, Adam Wagstaff, and Magnus Lindelow. 2008. *Analyzing Health Equity Using Household Data. A Guide to Techniques and Their Implementation*. Washington, DC: The World Bank.

Staiger, Douglas, and James H. Stock. 1997. "Instrumental Variables Regression with Weak Instruments." *Econometrica* 65, no. 3: 557-586.

Stock, James H., and Motohiro Yogo. 2005. *Testing for Weak Instruments in Linear IV Regression*. New York: Cambridge University Press.

WHO Multicentre Growth Reference Study Group. 2009. *WHO Child Growth Standards: Growth velocity based on weight, length and head circumference: Methods and development*. Geneva, Switzerland: World Health Organization.
